# Polymicrobial *Pasteurella multocida* ‐Anaerobic Coinfection Followhing a Cat Bite: Limb Salvage Through Metagenomic Next‐Generation Sequencing‐Guided Diagnosis and Multidisciplinary Management

**DOI:** 10.1002/ccr3.72304

**Published:** 2026-03-13

**Authors:** Yuanqing Qu, Yuan Liu, Xin Zhou, Pengjie Xu, Lu Wang

**Affiliations:** ^1^ Department of Laboratory Medicine The General Hospital of Western Theater Command Chengdu Sichuan China; ^2^ The Department of Clinical Laboratory at Hengyang Medical School University of South China (Associated With Nanhua Hospital) Hengyang Hunan China; ^3^ Department of Emergency Medicine The General Hospital of Western Theater Command Chengdu Sichuan China

**Keywords:** anaerobe, cat biting, metagenomics next‐generation sequencing (mNGS), mixed infection, *Pasteurella multocida*

## Abstract

Successful management of a *Pasteurella multocida* and polymicrobial infection following a cat bite on the left leg entailed debridement, split‐thickness skin grafting with vacuum‐sealing drainage, and targeted antibiotic treatment. This approach enabled successful incorporation of the skin graft, preserving the limb and eliminating the necessity for amputation.

## 1 Introduction

*Pasteurella* species, particularly *P. multocida* , are commonly implicated in infectious diseases in animals and humans, causing various infections such as wound infections, bloodstream infections, and osteomyelitis. Despite the extensive documentation of *P. multocida* infections, there is limited literature on deep local infections by this pathogen and the corresponding management strategies. In this study, we detected pathogenic bacteria in a wound with a mixed infection using conventional culture methods in combination with metagenomic next‐generation sequencing (mNGS). To prevent the progression to osteomyelitis, the patient underwent surgical interventions, including wound debridement, skin flap repair, and antibiotic therapy, which significantly contributed to a positive clinical outcome. This case report highlights the clinical presentation, management, and successful identification of *P. multocida* in a patient following a cat bite.

## 2 Case History/Examination

**FIGURE 1:**
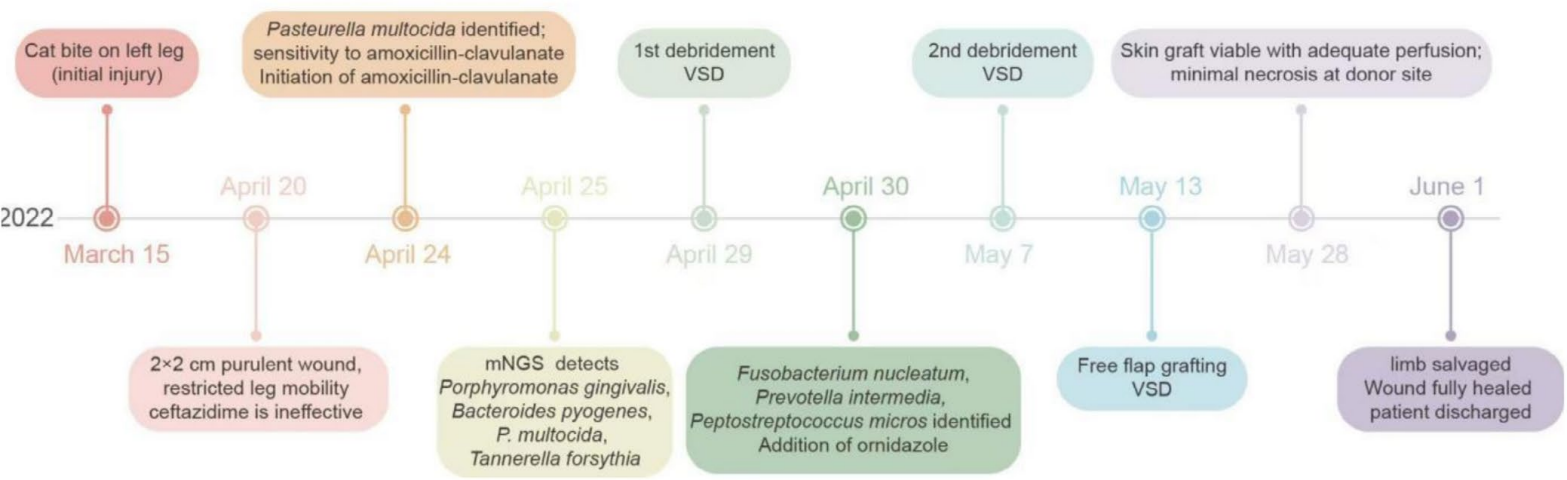
A timeline diagram of clinical events, featuring crucial elements such as the bite date, prior treatments, surgical interventions, and mNGS outcomes, and included it in the Case History section.

A timeline of clinical events (bite date, prior treatments, surgical interventions, and mNGS results) is provided in Figure 1 to help readers follow the clinical course more easily.

On April 20, 2022 (Day 1), a 56‐year‐old woman was hospitalized in the trauma center due to a skin and soft tissue infection caused by a cat bite on her left leg on March 15, 2022. Before admission, she had undergone several debridement interventions and had been treated with ceftazidime, amoxicillin‐clavulanate, or piperacillin‐tazobactam at a nearby medical facility. Nevertheless, the wound continued to exhibit yellow purulent discharge despite the administered treatments.

Upon admission, the patient exhibited a body temperature of 36.1°C (normal range: 36°C–37°C), a pulse rate of 92 beats/min (normal range: 60–100 beats/min), respiratory rate of 23 breaths/min (slightly above the normal range of 12–20 breaths/min), and blood pressure of 128/98 mmHg (normal range: 90–139/60–89 mmHg). Physical examination revealed minor swelling and minimal bleeding from the dressing on the left leg. A 2 × 2 cm wound with purulent discharge was observed, surrounded by multiple healed scratches, with tenderness at the wound margins. The left dorsalis pedis artery exhibited a palpable pulse, while mobility of the left leg was restricted. All other physical findings were within normal limits.

**FIGURE 2:**
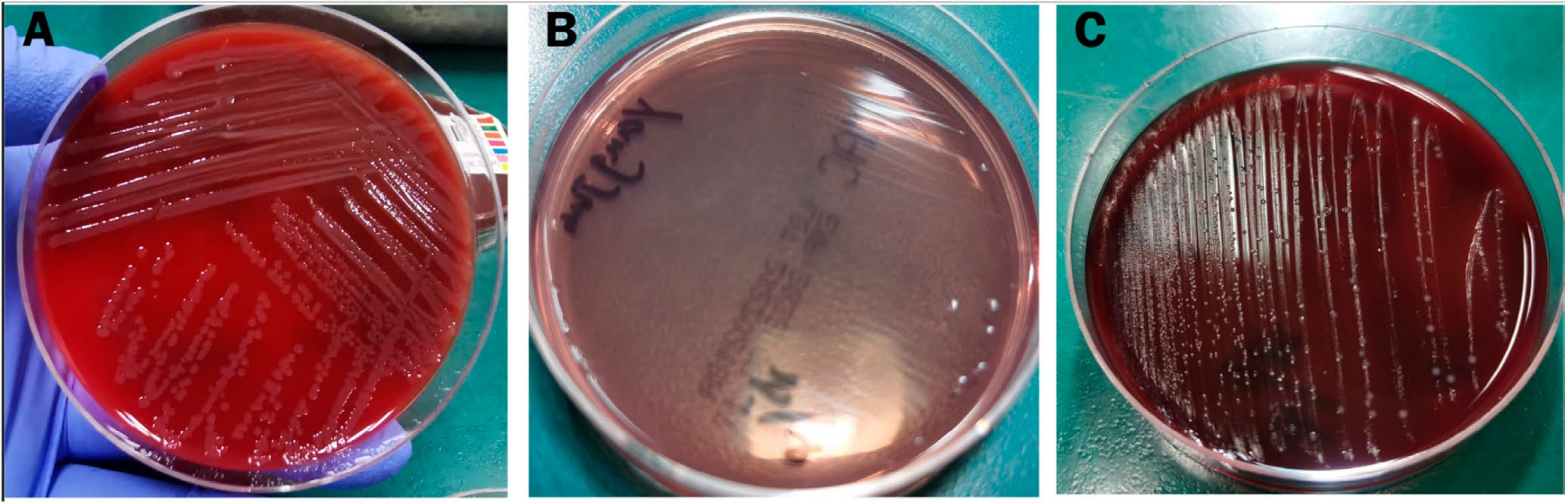
Culture outcomes of wound secretions: (A) Bacterial growth on Sheep Blood Agar by Day 4; (B) No growth on MacConkey Agar by Day 4 (characteristic of *P. multocida* ); (C) Bacterial growth on Brucella HK Medium by Day 10.

**FIGURE 3:**
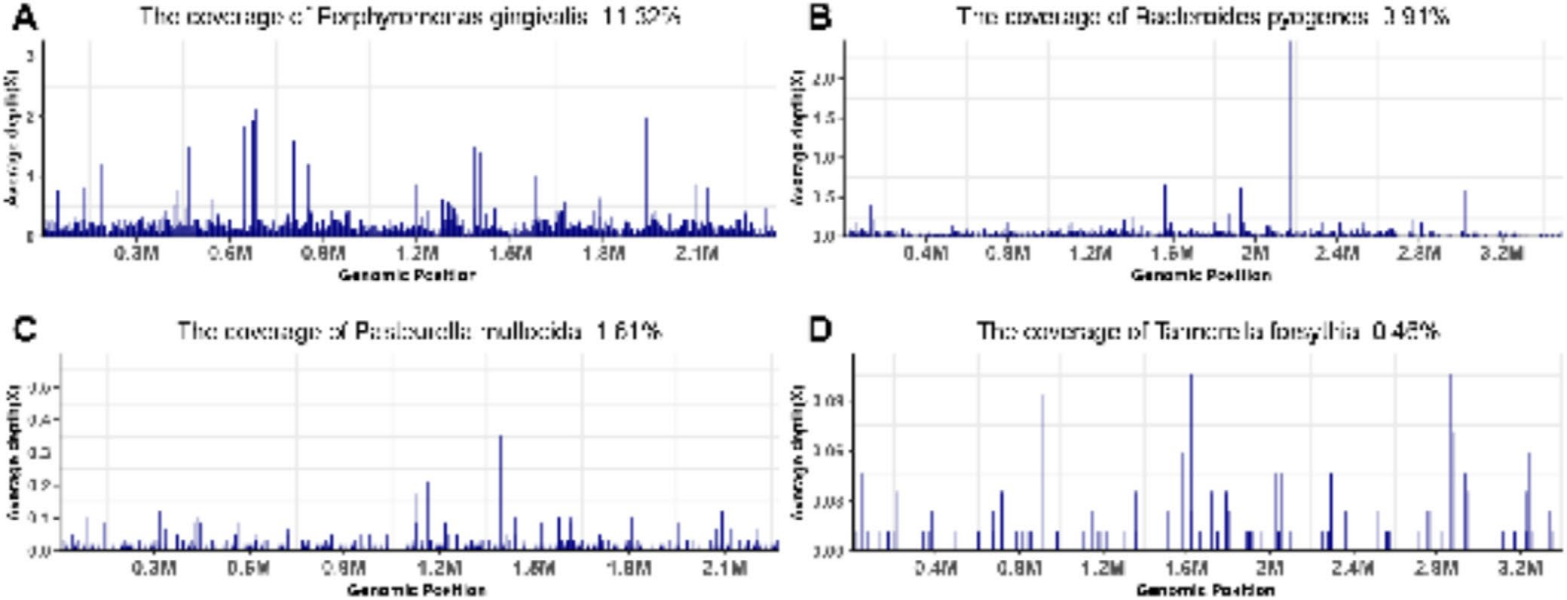
mNGS results (Day 5) showing genome coverages: *Porphyromonas gingivalis* (A, 11.32%), *Bacteroides pyogenes* (B, 3.91%), *Pasteurella multocida* (C, 1.61%), and *Tannerella forsythia* (D, 0.46%).

On the initial day (Day 1), wound secretion samples were collected for culture. Anaerobic culture samples were obtained on Day 1 and yielded positive results after 10 days of incubation. The wound secretion culture identified *P. multocida* by Day 4 (Figure 2) using VITEK 2 Compact Automatic Bacteria and Fungi Identification System (bioMérieux, France). Susceptibility testing on Day 5 indicated susceptibility to amoxicillin‐clavulanic acid, doxycycline, ampicillin, levofloxacin, and sulfamethoxazole‐trimethoprim (Table 1). Simultaneously, mNGS was performed on wound secretions, with results available by Day 5. The mNGS analysis used the Illumina MiSeq sequencing platform. The bioinformatics pipeline involved sequence alignment with BWA, microbial species annotation with Kraken2, and removal of host sequences (human genome hg38) to enhance the accuracy of the analysis. The analysis detected *Porphyromonas gingivalis* (873 sequence reads), *Bacteroides pyogenes* (4118 sequence reads), *P. multocida* (105 sequence reads), and *Tannerella forsythia* (114 sequence reads) (Figure 3), which were confirmed by anaerobic culture on Day 10. Species identification for these anaerobes was performed using VITEK 2 Compact Automatic Bacteria and Fungi Identification System (bioMérieux, France) combined with 16S rRNA gene sequencing. Porphyromonas species were not cultured despite high sequence reads detected by mNGS. This may be due to their strict requirements for growth conditions, as they need an exact anerobic environment and specific nutrients, while the anaerobic conditions during the primary culture process might not be ideal. In addition, factors such as DNA extraction efficiency, sequencing depth, and filtering thresholds also contribute to this phenomenon. Although Brucella HK medium contains hemin and vitamin K, other potential factors may contribute to growth failure, such as interspecies inhibition (e.g., short‐chain fatty acids produced by coexisting anaerobes suppressing its growth), slight medium drying during the 10‐day incubation reducing nutrient accessibility, or oxidative stress from transient exposure to oxygen during sample handling. Multiple bacterial species were identified from colonies grown on anaerobic media at Day 10, including *Fusobacterium nucleatum* , *Prevotella intermedia* , and *Peptostreptococcus micros* , and further identification tests using 16S rRNA gene sequencing were performed.

**TABLE 1:** Drug susceptibility tests of *Pasteurella multocida* .

Date	Antibiotic	Inhibition zone diameter (mm)	Minimal inhibitory concentration (μg/mL)	Sensitivity
Day 5	Levofloxacin	31	—	S
	Sulfamethoxazole‐trimethoprim	30	—	S
	Penicillin	20	—	I
	Ceftriaxone	30	—	I
	Erythromycin	16	—	R
	Amoxicillin‐clavulanic acid	29	—	S
	Doxycycline	30	—	S
	Ampicillin	28	—	S
Day 10	Ceftazidime	—	≤ 0.12	S
	Cefoperazone‐sulbactam	—	≤ 8	S
	Imipenem	—	≤ 0.25	S
	Tigecycline	—	≤ 0.5	S
	Levofloxacin	31	—	S
	Sulfamethoxazole‐trimethoprim	30	—	S
	Amoxicillin‐clavulanic acid	29	—	S
	Ampicillin	28	—	S
	Erythromycin	16	—	R
	Doxycycline	30	—	S

*Note:* Antimicrobial susceptibility testing was performed using the VITEK 2 AST‐GN card (bioMérieux, France). Interpretive criteria were based on CLSI M100 (32nd Edition). Isolates tested on Day 5 and Day 10 were subcultures of the same strain, confirmed by consistent 16S rRNA gene sequencing results.

Abbreviations: I, intermediate; R, resistant; S, susceptible.

**FIGURE 4:**
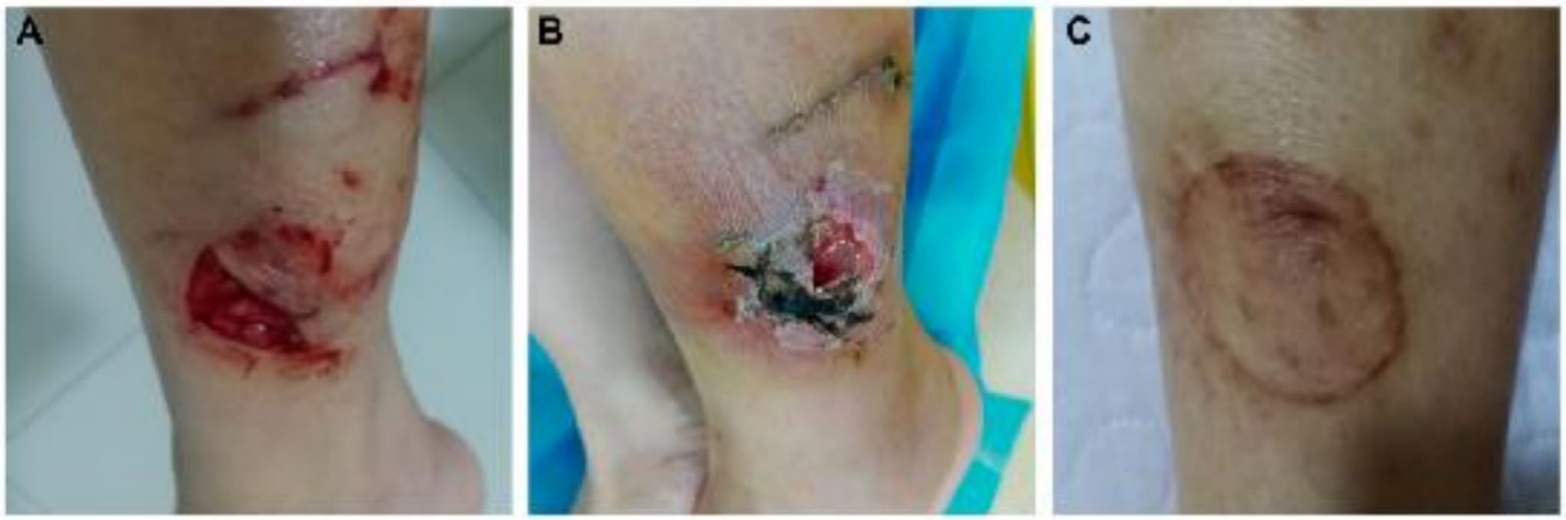
Wound progression: (A) March 15, 2022: 7 cm open, inflamed wound on the left calf with slight bleeding. (B) April 20, 2022: 2 × 2 cm wound with purulent discharge and surrounding scratches. (C) Current status: Satisfactory healing. Wound secretion culture findings: (A) Bacterial growth on Sheep Blood Agar (Day 4); (B) No growth on MacConkey Agar (Day 4), aiding initial identification of *P. multocida* ; (C) Growth on Brucella HK Medium (Day 10), confirming polymicrobial infection. mNGS identified obligate anaerobes ( *P. gingivalis* , *B. pyogenes* , *T. forsythia* ), which are difficult to culture aerobically, highlighting mNGS's diagnostic value. Multidisciplinary collaboration (microbiological diagnosis by the laboratory, surgical debridement, and wound care) was critical for limb salvage.

The patient underwent three surgical procedures at the Department of Burns and Plastic Surgery: on Days 9 and 17 (general anesthesia) for debridement of chronic ulcer and soft tissue infection, and vacuum‐sealing drainage (VSD); and on Day 23 (general anesthesia) for ulcer repair with free flap grafting and VSD. Postoperative anti‐infection therapy, guided by microbiological results (Table 1), included intravenous amoxicillin‐clavulanic acid (1.2 g every 8 h) and ornidazole (500 mg every 12 h). After 15 days, the skin graft area showed satisfactory healing with adequate perfusion, minimal necrosis, and slight bleeding at the donor site (left thigh). The patient was discharged on Day 42 with well‐healed wounds (Figure 4).

## 3 Differential Diagnosis, Investigations, and Treatment

The differential diagnosis focused on accurately identifying the pathogen. The local county hospital initially misidentified *P. multocida* and anaerobes as *Escherichia coli* , leading to inappropriate antibiotic use. The misidentification might be attributed to two reasons: first, the growth morphology of the bacteria was similar during primary culture, and the local laboratory lacked specific identification tests for fastidious bacteria; second, the patient had received empirical antimicrobial treatment before the clinical specimen was submitted for testing, which might have inhibited the growth of the bacteria, thereby affecting their isolation and identification. In contrast, our laboratory performed bedside inoculation and expedited mNGS, with susceptibility results (Table 1) guiding targeted therapy. mNGS served as a valuable supplementary diagnostic tool.

## 4 Conclusion and Results (Outcome and Follow‐Up)

See Figures 1, 2, 3, 4 and Table 1.

## 5 Discussion

*P. multocida* , a zoonotic bacterium, predominantly inhabits the respiratory and intestinal tracts of various domestic and wild animals. Human infections commonly arise from scratches and bites by animals such as cats, dogs, or other species [1]. Animal bites represent a significant global public health issue affecting individuals of all ages [2]. In China, approximately 40 million people sustain cat and dog bites annually [3, 4]. Cat bites, accounting for 2%–50% of all animal bites worldwide [2], pose a higher infection risk compared to dog bites [5]. Notably, *P. multocida* is the primary pathogen identified in cat bite wounds, known to possess various virulence factors such as capsular lipopolysaccharide, cytotoxin, adhesin, and hemagglutinin, which can lead to diverse complications [6, 7, 8]. Following a scratch or bite, initial symptoms like wound swelling, pain, and purulence appear, with deeper wounds potentially causing periostitis, osteomyelitis, and sepsis. Cat bites, in particular, can result in deep abscesses and osteomyelitis due to the penetrating nature of feline teeth [9]. Additionally, inhalation of *P. multocida* ‐containing aerosols can lead to respiratory tract infections [10].

Approximately 35% of cat bite patients were found to be infected with *P. multocida* [11], with a majority of cases involving polymicrobial infections [9, 10, 11, 12]. Anaerobes (*Porphyromonas*, *Bacteroides*) account for > 60% of isolates [6, 8, 9, 10, 11, 12, 13].

*P. multocida* is a facultative anaerobe and grows well in standard aerobic culture conditions. However, other anaerobes (e.g., *Porphyromonas*) are risking underdiagnosis, which presents challenges in detection due to its strict culture requirements and slow growth rate. [14]. Persistent and worsening skin and soft tissue infections following animal scratches can result in secondary osteomyelitis. Successful management in our case involved thorough debridement, vacuum‐sealed drainage (VSD), flap repair, and antimicrobial therapy. VSD is effective in managing chronic wound infections by controlling the infection, improving wound perfusion, and safeguarding vital structures like blood vessels, nerves, and tendons. Skin flap transplantation is a valuable strategy for treating infected wounds, restoring blood supply, and resolving chronic infections [15]. Our investigation identified *P. multocida* and anaerobic bacteria through both culture and mNGS. Timely surgical interventions, including debridement, VSD utilization, flap repair, and antibiotic treatment, were crucial in halting the progression of the wound infection to osteomyelitis. Ultimately, the patient was discharged with a favorable prognosis.

The popularity of pet ownership has increased with rising living standards, leading to a higher incidence of cat or dog scratches and bites. Timely wound debridement and anti‐infective therapy are vital in managing such incidents. There is currently no clinical vaccine available for bite‐related polymicrobial infections [16], such as those caused by *P. multocida* and *Porphyromonas* species, and the previous statement about potential bacteria preventable by vaccination was inaccurate, which is hereby clarified. Guidelines for *P. multocida* are lacking, but penicillin, ampicillin, and piperacillin are recommended [10, 17].

Polymicrobial infections commonly arise postanimal bites, often posing challenges for conventional microbiological testing due to limited pathogen identification. mNGS presents a promising solution by enabling direct detection of all microbial nucleic acids from clinical samples, bypassing the need for culture. This approach offers enhanced detection rates, sensitivity, and expedited results, particularly advantageous for uncovering rare, unculturable, and polymicrobial infections. The integration of mNGS with traditional diagnostic techniques is recommended to optimize clinical outcomes [18]. However, mNGS has certain limitations, including high cost, limited availability in some regions, relatively long turnaround time (72 h in this case), and restricted applicability in resource‐limited settings due to the lack of necessary equipment and technical personnel. While mNGS facilitates comprehensive microbial profiling, clinicians must prioritize critical pathogens to enhance clinical applicability [19]. Moreover, timely and appropriate interventions, including surgical debridement and antimicrobial therapies, are imperative for effectively managing these intricate infections.

## Author Contributions

**Yuanqing Qu:** conceptualization, data curation, investigation, methodology, project administration, resources, software, supervision, validation, visualization, writing – original draft, writing – review and editing. **Yuan Liu:** conceptualization, data curation, formal analysis, funding acquisition, investigation, methodology, project administration, resources, supervision, visualization, writing – original draft, writing – review and editing. **Xin Zhou:** conceptualization, data curation, formal analysis, investigation, resources, software, validation, visualization, writing – original draft, writing – review and editing. **Pengjie Xu:** conceptualization, data curation, formal analysis, methodology, project administration, validation, visualization, writing – original draft, writing – review and editing. **Lu Wang:** conceptualization, data curation, formal analysis, funding acquisition, investigation, methodology, project administration, resources, software, supervision, validation, visualization, writing – original draft, writing – review and editing.

## Funding

This work was supported by Hospital Management Project of General Hospital of Western Theater Command (2021‐XZYG‐B11).

## Ethics Statement

This study received approval from the General Hospital of Western Theater Command of PLA. The patient provided written informed consent for participation and publication of any potentially identifiable images or data in this case report. All original data are included in the article, with further inquiries directed to the corresponding author. The authors report no conflicts of interest. Funding was provided by the Hospital Management Project of General Hospital of Western Theater Command (2021‐XZYG‐B11).

## Consent

Written informed consent was obtained from the patient for publication of this case report and accompanying images, complying with the requirements as mentioned in Wiley's CCR Consent Form.

## Conflicts of Interest

The authors declare no conflicts of interest. The study's original contributions are detailed in the article; additional inquiries should be directed to the corresponding author.

## Data Availability

The authors have nothing to report.
